# Audiovestibular Dysfunction in Hyper-IgE Syndrome: A Systematic Review of Characteristics, Pathophysiology, Diagnosis, and Management

**DOI:** 10.3390/ijms26209932

**Published:** 2025-10-12

**Authors:** Jiann-Jy Chen, Chih-Wei Hsu, Brendon Stubbs, Tien-Yu Chen, Chih-Sung Liang, Yen-Wen Chen, Bing-Yan Zeng, Ping-Tao Tseng

**Affiliations:** 1 Prospect Clinic for Otorhinolaryngology & Neurology, Kaohsiung 81166, Taiwan; jiannjy@yahoo.com.tw (J.-J.C.); kevinachen0527@gmail.com (Y.-W.C.); 2 Department of Otorhinolaryngology, E-Da Cancer Hospital, I-Shou University, Kaohsiung 82445, Taiwan; 3 Department of Psychiatry, Kaohsiung Chang Gung Memorial Hospital and Chang Gung University College of Medicine, Kaohsiung 833401, Taiwan; harwicacademia@gmail.com; 4 Department of Psychological Medicine, Institute of Psychiatry, Psychology and Neuroscience, King’s College London, London WC2R 2LS, UK; brendon.stubbs@kcl.ac.uk; 5 Department of Sport, University of Vienna, 1010 Vienna, Austria; 6 Department of Psychiatry, Tri-Service General Hospital, Taipei 114202, Taiwan; verducciwol@gmail.com; 7 Department of Psychiatry, College of Medicine, National Defense Medical University, Taipei 114202, Taiwan; 8 Department of Psychiatry, Beitou Branch, Tri-Service General Hospital, School of Medicine, National Defense Medical University, Taipei 11243, Taiwan; lcsyfw@gmail.com; 9 Department of Psychiatry, National Defense Medical University, Taipei 114202, Taiwan; 10 Institute of Biomedical Sciences, National Sun Yat-sen University, Kaohsiung 80424, Taiwan; 11 Department of Internal Medicine, E-Da Dachang Hospital, I-Shou University, Kaohsiung 807066, Taiwan; 12 Department of Psychology, College of Medical and Health Science, Asia University, Taichung 406040, Taiwan; 13 Institute of Precision Medicine, National Sun Yat-sen University, Kaohsiung 80424, Taiwan

**Keywords:** Hyper-IgE syndrome, cochleopathy, vestibular, sensorineural hearing loss, treatment

## Abstract

Hyper-IgE syndrome (HIES) is a rare genetic immunodeficiency characterized by elevated serum IgE levels and associated immune dysregulation, manifesting in recurrent infections, eczema, and skeletal abnormalities. Emerging evidence suggests a link between HIES and audiovestibular dysfunction, potentially mediated by IgE-driven inflammation in the inner ear, which is not immunologically privileged. However, the nature of this association remains poorly understood. This systematic review synthesizes current evidence on the characteristics, pathophysiology, diagnostic approaches, and management of audiovestibular dysfunction in HIES patients. Literature searches across PubMed, Embase, ClinicalKey, Web of Science, and ScienceDirect (up to 6 August 2025) were conducted in accordance with PRISMA guidelines. Key findings indicate that HIES-related audiovestibular issues, including sensorineural hearing loss and vestibular impairment, may arise from IgE-mediated endolymphatic sac inflammation, leading to hydrops and hair cell damage. Diagnostic tools such as audiometry, electrocochleography, and allergen challenge tests show promise, with elevated IgE correlating with abnormal otoacoustic emissions and prolonged auditory brainstem response latencies. Treatment focuses on immunomodulation (e.g., corticosteroids, dupilumab) to mitigate IgE effects, though evidence is limited to case reports. A proposed schematic diagram illustrates pathophysiology, emphasizing IgE’s role in inner ear toxicity. Timely recognition and intervention may prevent progression to permanent hearing loss or vestibular disability, improving quality of life. Future research should explore genetic–immunologic mechanisms and prospective trials for targeted therapies. Trial registration: PROSPERO CRD420251120600.

## 1. Introduction

Hyper-IgE (immunoglobulin E) syndrome, also known as Job’s syndrome, is a rare genetic immunodeficiency characterized by elevated serum IgE levels, recurrent infections, eczema, and skeletal abnormalities [1]. Hyper-IgE syndrome was first described by Davis and colleagues [2] and further characterized by Buckley and colleagues [3]. Hyper-IgE syndrome is a rare disease and accurate epidemiological data on its prevalence rates remain limited. Hyper-IgE syndrome can affect individuals of all ethnicities worldwide. Hyper-IgE syndrome has an estimated incidence of 1:1,000,000, affecting both sexes equally [4], and is characterized by a high variety of clinical manifestations resulting from multiple defects in both innate and adaptive immunity [5]. No specific epidemiological data exist for hyper-IgE syndrome-related audiovestibular dysfunction. Indirect evidence from related conditions shows 50% of Ménière’s disease patients report allergies, with 25.8% exhibiting elevated IgE levels, though few reach hyper-IgE syndrome thresholds [6,7].

No studies directly link hyper-IgE syndrome genetic deficits (e.g., STAT3 mutations) to audiovestibular dysfunction. Rather, the potential linkage between hyper-IgE syndrome and audiovestibular dysfunction might rely on its abnormal IgE levels. In theory, the overtly elevated IgE levels play an important role in the disturbed function of the audiovestibular system [8], which is associated with the sinuses, bones, and respiratory system [9,10]. Emerging evidence links immune-mediated diseases, including systemic lupus erythematosus [11], rheumatoid arthritis [12], anti-phospholipid syndrome [10], Hashimoto’s disease [13], thyroid dysfunction [14], and allergic inflammation [9], to inner ear pathology, raising the hypothesis that dysregulated IgE in hyper-IgE syndrome could contribute to audiovestibular impairment [15]. Along with the increased recognition of this disease, the definition of hyper-IgE syndrome has now been expanded to represent a category of three main features (i.e., overtly increased IgE levels, high susceptibility to infection, and unexplainably recurrent allergic reaction), and is not limited to cases with genetically abnormal findings in a gene examination [16]. Although there might be some diversity in different definitions across different medical societies, the main core of hyper-IgE syndrome still relies on its three main clinical features.

The inner ear is not immunologically privileged [17], with the endolymphatic sac acting as an immune-active site that secretes immunoglobulins and responds to cytokines [18,19]. Theoretically, the immunologically active endolymphatic sac would react to cytokines, such as IgE, and lead to influx of fluid with potassium and subsequent toxicity [20] so that it might finally result in audiovestibular dysfunction in the affected inner ear [21]. Elevated IgE levels, as seen in hyper-IgE syndrome, may trigger inflammation, leading to endolymphatic hydrops, a disorder which is characterized by an abnormal accumulation of endolymph and results in increased inner ear pressure and distortion, and sensorineural hearing loss [22,23]. In a case-control study investigating pure-tone audiometry outcomes in subjects with elevated IgE levels, the authors noticed a phenomenon of a higher prevalence of high-frequency sensorineural hearing loss for pure-tone audiometry and abnormal otoacoustic emissions in allergic patient groups than in controls [24]. Epidemiological studies report a 41.6% prevalence of airborne allergies in Ménière’s disease, with higher IgE levels correlating with audiovestibular dysfunction, often bilateral, compared to controls [20,21,25]. In a previous retrospective report, researchers noticed that allergic reactions in patients were associated with audiologic consequences (50.5%), vestibular consequence (33.7%), Ménière’s disease (9.5%), and overall autoimmune inner ear disease (6.3%) [26]. Most cases were bilaterally involved rather than unilaterally affected [25]. High levels of peripheral total and specific IgE were noticed in patients with Ménière’s disease compared to controls, with levels equivalent to those noted for allergic reaction [21]. Unlike idiopathic hearing loss, hyper-IgE syndrome-related audiovestibular dysfunction may be reversible with timely intervention, highlighting the need for early detection [27].

Despite these insights, evidence on hyper-IgE syndrome’s audiovestibular effects is sparse, limiting clinical management. This systematic review aims to synthesize current knowledge on the characteristics, pathophysiology, diagnostic approaches, and treatment of audiovestibular dysfunction in patients with hyper-IgE syndrome, providing clinicians with evidence to guide practice and future research.

## 2. Methods

### 2.1. Study Design and Registration

**Figure 1:**
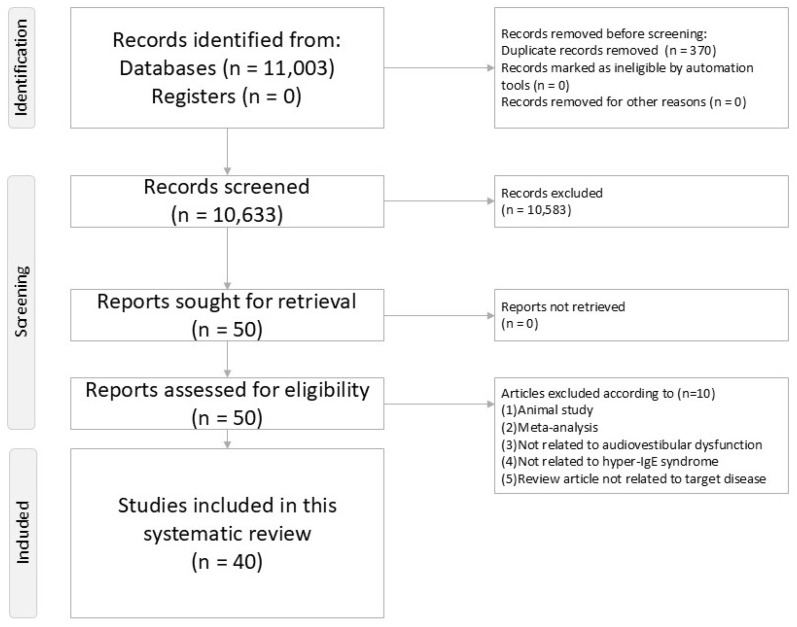
PRISMA2020 flowchart of current systematic review.

This systematic review adhered to the Preferred Reporting Items for Systematic Reviews and Meta-Analyses (PRISMA) guidelines [28] and was registered with PROSPERO (CRD420251120600). A PRISMA checklist is provided (Table S1) and study selection is illustrated (Figure 1).

Figure 1 demonstrates the flowchart illustrating the procedure of the present systematic review.

### 2.2. Search Strategy

We searched PubMed, Embase, ClinicalKey, Web of Science, and ScienceDirect from inception to 6 August 2025, using keywords and MeSH terms related to hyper-IgE syndrome and audiovestibular dysfunction (Table S2). Manual searches of reference lists from included studies and relevant reviews supplemented the electronic search. No language or publication date restrictions were applied. Corresponding authors were contacted for additional data when necessary. We did not set strict limitations regarding the operational definition of hyper-IgE syndrome due to the fact that the definition of hyper-IgE syndrome might vary across different regions and time periods due to lack of consensus currently. Further, we did not set any restriction on the publication date during our search process. We did not set restrictions on any language of publication. If any papers were published in a language other than English or Chinese, we referred to some translator tools, such as Google translator.

### 2.3. Eligibility Criteria

Although it would be helpful to set specific definition of hyper-IgE syndrome, it would be difficult because there has been no conclusive operational definition of such diseases due to its rarity [1]. Inclusion criteria were: (1) studies addressing characteristics, pathophysiology, diagnosis, or treatment of audiovestibular dysfunction in patients with hyper-IgE syndrome; (2) study designs including case reports, case series, observational studies, case-control studies, or randomized controlled trials; (3) studies involving patients diagnosed with hyper-IgE syndrome.

Exclusion criteria were: (1) studies not involving hyper-IgE syndrome patients; (2) studies unrelated to audiovestibular dysfunction characteristics, pathophysiology, diagnosis, or treatment; (3) animal studies.

Excluded articles are listed in Table S3.

### 2.4. Screening and Selection

Two authors (P.-T.T., Y.-W.C.) independently screened titles and abstracts from all databases, followed by full-text review of eligible articles. Duplicates were removed manually using reference management software. Discrepancies were resolved through discussion or consultation with a third author (J.-J.C.).

### 2.5. Data Extraction

Two authors (P.-T.T., Y.-W.C.) independently extracted data on study characteristics (e.g., design, sample size), patient demographics, audiovestibular dysfunction characteristics, pathophysiology, diagnostic methods, and treatments from full texts. Discrepancies were resolved through consensus.

### 2.6. Quality Assessment

Study quality was independently assessed by P.-T.T. and Y.-W.C. using the Newcastle–Ottawa Scale for non-randomized studies [29] or the Cochrane Risk of Bias tool for randomized trials, if applicable (Table S4). Discrepancies were resolved through discussion. Quality scores informed narrative synthesis but did not influence inclusion.

### 2.7. Data Synthesis

Data were synthesized narratively due to anticipated heterogeneity in study designs and outcomes. Findings were categorized by characteristics, pathophysiology, diagnosis, and treatment of audiovestibular dysfunction in hyper-IgE syndrome. Quantitative meta-analysis was not planned due to expected variability in study methodologies and reporting. However, meta-analysis was considered if there were ≥3 sufficiently homogeneous datasets available for the same outcome.

## 3. Results

### 3.1. Study Selection

Following PRISMA guidelines, our search (up to 6 August 2025) yielded limited studies directly addressing audiovestibular dysfunction in hyper-IgE syndrome after excluding ineligible studies (Table S3). Most evidence was derived from case reports, observational studies, and related immune-mediated conditions (e.g., Ménière’s disease, allergic rhinitis). Study characteristics and quality assessments are detailed in Tables S4 and S5.

### 3.2. Characteristics and Epidemiology

Hyper-IgE syndrome is a rare primary immunodeficiency [30], which consists of three major features, including (1) hyper-elevated serum IgE levels, (2) enhanced susceptibility to any types of infection, and (3) eczema/atopic dermatitis/asthma bronchiale/allergic reaction, which we called the hyper-IgE syndrome symptomatic triad [16]. The incidence of this disease is less than 1 in 1,000,000 people [31]. Further, the incidence of hyper-IgE syndrome is about 6 to 10 cases per year [32]. Hyper-IgE syndrome is considered to be a genetic disease, resulting from either de novo genetic changes or autosomal recessive/dominant inheritance [33]. The overt reaction to allergens via elevated IgE expression is one of the major features of hyper-IgE syndrome [33]. Patients with hyper-IgE syndrome were reported to have higher lifetime prevalence rates and higher severity of allergy reaction than healthy controls [33]. However, all the prevalence data might be overly conservative because under-diagnosis of hyper-IgE syndrome occurs frequently due to its similar presentation with common atopic diseases in their early stages [5].

### 3.3. Pathophysiology

While the genetic basis (e.g., STAT3, DOCK8, PGM3, or ZNF341 mutations) [34,35,36] and systemic manifestations of hyper-IgE syndrome are well-documented [37,38,39], its association with audiovestibular dysfunction remains underexplored. In some patients with a specific genetic mutation (i.e., STAT3), there was inflammation-mediated demyelination and astrocytosis noticed in brain images in patients with hyper-IgE syndrome [40]. In addition to the aforementioned IgE and related cytokine-induced central nervous system damage, previous reports have demonstrated the possibility of chronic inflammatory demyelinating polyneuropathy in a patient with hyper-IgE syndrome [41]. Therefore, the cochlear nerve might also serve as potential target of such diseases through the potential linkage of immune-mediated vasculitis [1]. Renner et al. demonstrated a potential linkage between nerve damage and IgE-associated vasculitis [42]. Elevated IgE levels, as seen in hyper-IgE syndrome, may trigger inflammation, leading to endolymphatic hydrops, a disorder that is characterized by an abnormal accumulation of endolymph and results in increased inner ear pressure and distortion, and sensorineural hearing loss [22]. In addition to the overtly increased IgE, there were several proinflammatory cytokine over-productions noticed in patients with hyper-IgE syndrome, which indicated the fact that the features of hyper-IgE syndrome were a mixed autoimmune storm with multiple organs involved [43]. Beyond the IgE over-production, the dysfunctional JAK-STAT pathways, interleukins 6 and 17, and Th17 lymphocytes also contributed to the multi-organ damage features of hyper-IgE syndrome [44].

**Figure 2:**
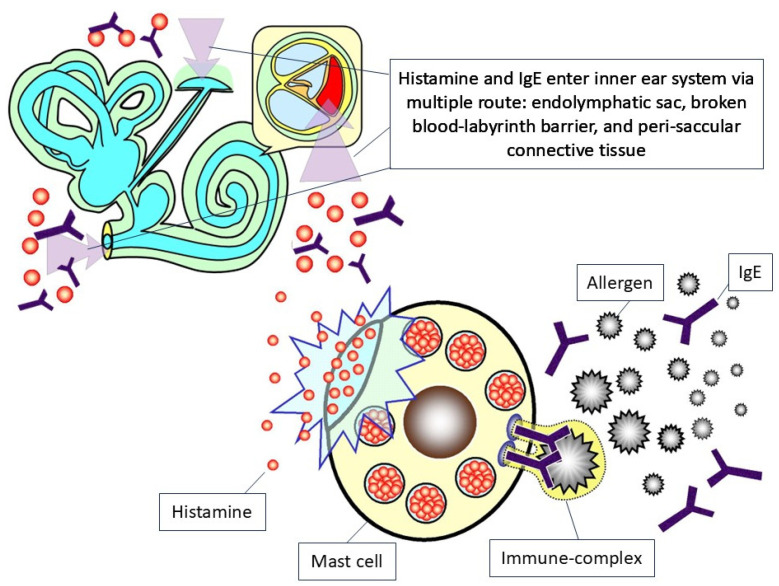
Schematic diagram of the physiopathology of hyper-IgE syndrome in audiovestibular dysfunction.

Another potentially direct linkage between genetic deficits and audiovestibular dysfunction might come from the facial deformity related to a genetic deficit [45]. Specifically, previous reports summarized some hyer-IgE syndrome-associated comorbidities, including skin abscesses, severe sinusitis, osteomyelitis, and pneumonia [46]. In the case report by Borst et al. [47], the authors demonstrated a recurrent pneumonia and sinusitis related to hyper-IgE syndrome-related deformities. Theoretically, the hyper-IgE levels would lead to complicated sinusitis [48]. In some severe forms of hyper-IgE syndrome-related infection, it might lead to facial structure perforation and consequent complications [49]. The allergic rhinosinusitis related to hyper-IgE syndromes [50] might be a predisposal factor in consequent audiovestibular impairment [9]. The nasal polyposis, a subtype of chronic rhinosinusitis related to hyper-IgE syndrome deformities [51], might lead to mucosa obstruction and consequent local inflammation within the facial region [9]. In summary, Figure 2 depicts the possible physiopathology linkage between hyper-IgE syndrome and inner ear impairment.

Figure 2 illustrates the physiopathology of hyper-IgE syndrome associated with audiovestibular dysfunction. It overall consists of several steps, including (1) the combination of IgE and allergen contributes to histamine secretion from the mast cell, (2) the IgE and histamine enter the inner ear system via the routes of endolymphatic sac, broken blood–labyrinth barrier, and peri-saccular connective tissue, and (3) finally lead to the inflammation process and consequent destruction of the inner ear system.

### 3.4. Diagnostic Approaches

In patients with hyper-IgE syndrome, the serum IgE levels might achieve levels as high as ten times greater than the normal limits of healthy subjects [52]. In some rare conditions, the IgE levels could achieve levels as high as 2000 to 80,000 IU/mL in patients with hyper-IgE syndrome [53]. In a previous report by and the colleagues [22], the authors demonstrated that the cutoff point of IgE (i.e., total IgE: 177.42 IU/mL, specific IgE 3.53 IU/mL) could serve as an indicator of transformation of Ménière’s disease and the initiation of sensorineural hearing loss. Further, the total IgE exhibited a better predictive ability due to the greater area under the curve for total IgE in their study [22]. The overtly increased IgE levels were considered to be the main feature of this syndrome. Therefore, in situations of suspected hyper-IgE syndrome, the IgE levels should help in the early detection of hyper-IgE syndrome [54].

### 3.5. Treatment

Lan and colleagues applied omalizumab, a humanized recombinant monoclonal antibody against IgE, to manage the complication of hyper-IgE syndrome in an autosomal dominant hyper-IgE syndrome patient [55]. In their report, the patient responded well and remained healthy with stable IgE levels during the one-year follow up [55]. In some cases with severe complications, intravenous immunoglobulin (IVIG) might be effective in immune disease treatment [56]. Some reports recommended a combination of maintenance of IVIG and prophylactic antibiotics in some refractory cases [57]. Antiseptic therapies for the skin such as chlorhexidine might also have merit in infection prevention [58]. In addition, antifungal prophylaxis might also be considered in some cases with high susceptibility to fungal infection [59].

However, in rare cases, hematopoietic cell transplantation might be considered as an alternative treatment option in STAT3-associated hyper-IgE syndrome patients in severe situations [60,61]. The hematopoietic cell transplantation, although it could not alter the nature of genetic mutations, could help in the restoration of cytokine production and reduce the frequency of infection in long-term follow up [62].

### 3.6. Prognosis

Although the IgE-related complications might be relatively mild and less life-threatening, in some rare conditions, hyper-IgE syndrome might be associated with encephalomyelitis, which is a life-threatening emergency [63]. Specifically, the immunodeficient status related to hyper-IgE syndrome would limit the protectivity of these patients and make them prone to opportunistic infection and consequent central nervous system infection [64]. According to the report by Freeman and colleagues, the most important risk factor leading to mortality in hyper-IgE syndrome was the respiratory failure that resulted from the frequent respiratory system infections [65], and the respiratory system is highly associated with the inner ear system.

## 4. Discussion

This systematic review synthesizes the sparse evidence on audiovestibular dysfunction in hyper-IgE syndrome, revealing a potential yet underexplored link mediated by elevated IgE levels and immune dysregulation. While direct studies are lacking, indirect insights from allergic and immune-mediated conditions suggest IgE-driven inflammation in the endolymphatic sac as a central mechanism, leading to hydrops, hair cell damage, and sensorineural hearing loss [8,22]. Increased CD4/CD8 ratios and CD23 levels enhance IgE transport, exacerbating cochlear and vestibular damage [21].

Additionally, the fenestrated vascular structure of the sac facilitates allergen and IgE entry, triggering mast cell degranulation and cytokine cascades that disrupt cochlear and vestibular function [26,66]. Specifically, the allergens could easily enter the peripheral blood vessels of the endolymphatic sac because of their fenestrated structure [67], which would lead to mast-cell degranulation in the connective tissue around the endolymphatic sac [66]. Although for the most part the labyrinth is protected by a blood–labyrinthine barrier, the fenestrated structure of the posterior meningeal artery would serve as an entrance for extrinsic allergens [27]. On the other hand, the inflammation-mediated demyelination and astrocytosis noticed in brain images either in astrocyte-specific Stat3 knockout mice [68] or in patients with Stat3 mutation hyper-IgE syndrome [40] would also support the hypothesis of central nervous inflammation theory.

Genetic factors in hyper-IgE syndrome, such as STAT3 mutations, may exacerbate this through impaired immune regulation, though facial deformities and sinusitis offer secondary pathways [1,9]. As addressed before, the allergic microbial rhinosinusitis related to hyper-IgE syndromes [50] might be a predisposal factor of consequent audiovestibular impairment [9]. Specifically, the sinusitis and consequent local inflammation within the facial region [9], which may result from anatomical deformity in hyper-IgE syndrome [1], would serve as one of the major origins of audiovestibular dysfunction according to our previous report [9]. These mechanisms align with observations in Ménière’s disease and allergic rhinitis, where IgE correlates with bilateral involvement and high-frequency sensorineural hearing loss [24,25].

In addition to direct observation in clinical reports of hyper-IgE syndrome, the clinical observations from allergy studies could also serve as references of physiopathology of hyper-IgE syndrome in audiovestibular dysfunction. Specifically, the exposure to allergens and consequent IgE increase not only came from the peripheral blood but also from direct air exposure. Specifically, as we know, the inner ear is not completely isolated from the air tract. The inhaled allergens, such as mugwort and ragweed, could have an impact on middle ear mucosa via the eustachian tube, leading to subsequent sensorineural hearing loss. Besides, due to the high permeability of the round window membrane, several inflammatory cytokines and IgE could penetrate into the inner ear structure easily and contribute to consequent conductive hearing loss. After IgE enters the inner ear system, it would trigger the downstream immune reaction mediated by mast cells and basophils and lead to inner ear system damage [69]. After sensitization, the overt IgE-mediated degranulation of the mast cells would lead to the infiltration of eosinophilia in the peri-saccular connective tissue and the clinical production of endolymphatic hydrops [66]. The locally increased inflammatory mediator and toxic accumulation of metabolic products in the inner ear system would disrupt hair cell function [26]. Additionally, the penetrated IgE would lead to endolymphatic hydrops formation in the endolymphatic sac and disturb the operating point of the cochlear microphonic, producing a direct current (DC) offset and thereby elevating the cochlear summating potential (SP)/auditory nerve action potential (AP) ratio [70].

Diagnostic challenges persist, with elevated IgE thresholds (>177.42 IU/mL) predicting progression, but reliance on audiometry, electrocochleography, and allergen challenges limits specificity in hyper-IgE syndrome [19,22]. Treatment remains empirical, focusing on immunomodulation with steroids or biologics like dupilumab, showing promise in reducing inflammation but lacking hyper-IgE syndrome-specific trials [71,72]. Prognosis suggests a fluctuating course due to persistent IgE memory, underscoring the need for early intervention to prevent irreversible impairment [73]. In some advanced cases, empirically prophylactic antibiotics should be considered to preserve audiovestibular function in hyper-IgE syndrome with refractory sinusitis [74].

Regarding the diagnosis of audiovestibular dysfunction related to hyper-IgE syndrome, the exacerbated IgE raised after allergen stimulation during the course of hyper-IgE syndrome related to audiovestibular disorders. Therefore, researchers tried to use allergen challenge tests and consequent change in electrophysiological tests as a diagnostic tool. Specifically, Clemis et al. [75] and Gibbs et al. [19] placed allergens in patients’ mucosa to challenge their IgE levels and recorded the changes of electronystagmography and electrocochleography, hoping to represent the vestibule and audiology dysfunction related to abnormal IgE levels. Finally, the serum immunoreactivity to allergen stimulation would have a significant relationship to Ménière’s disease [76]. In addition to the electrophysiology study, audiological examinations would have merit in the diagnosis of hyper-IgE-related inner ear diseases. Specifically, in subjects with elevated IgE levels, they would have significantly abnormal transient evoked otoacoustic emission (TEOAE) and distortion product otoacoustic emission (DPOAE) findings in frequency of 1, 1.5, 2, 3, and 4 kHz compared to controls [77], which reflects the potential outer hair cell dysfunction in such subjects. In addition, the auditory brainstem response study also revealed a statistically significant difference in some wave latencies and interpeak latencies [77]. Specifically, a prolongation of wave I latency and shortening of waves I–III and I–V interpeak latencies was noticed, which indicated cochlear involvement in those subjects [78]. Furthermore, in subjects with elevated IgE levels, there was a statistically significant increase in the pure-tone threshold at 4000 Hz [24] to 8000 Hz [79]. Additionally, 18% subjects would also display conductive hearing loss in their audiogram study [24]. Although researchers have proposed a radio-allergosorbent test to help in the detection of immune reactions within the inner ear and eustachian tube [80], this should be used with special caution due to the risk of radiation exposure.

In ordinary clinical settings, it would be difficult distinguishing hyper-IgE syndrome patients from common allergic patients, especially in their early disease stage. Although a detailed family history and clinical course might help in differential diagnosis, specific laboratory tests for hyper-IgE syndrome would be strongly recommended. Since hyper-IgE syndrome is frequently complicated with multi-organ immune-mediated damage, early detection of hyper-IgE syndrome is especially important to protect patients’ end organs. Finally, to deeply realize the nature of hyper-IgE syndrome in specific patients, genetic testing panels are warranted [16].

There has not been clear evidence regarding treatment directly targeting audiovestibular dysfunction related to hyper-IgE syndrome. The most evidence mainly came from treatment for IgE-related immune reactions [31]. First of all, although IgE was considered to be associated with allergic reaction, this did not guarantee the efficacy of anti-allergic medication for IgE-related sensorineural hearing loss [22]. This was because the IgE re-stabilization would restore the depleted peripheral IgE levels rapidly when anti-allergic medication was prescribed [81]. Rather, medications targeting IgE plus memory B cells, such as eculizumab, might be effective in reducing IgE levels continuously [82]. Similarly, in a case report, the authors noticed that dupilumab, an anti-IL-4 receptor agent, could restore patients’ hearing and reduce tinnitus severity based on the theory that IgE levels in middle ear effusion could correlate with hearing dysfunction [72]. On the other hand, interferon-γ therapy might be helpful in the management of excess-IgE-related diseases [71]. Finally, along with its immunological nature, the prescription of steroid treatment might help to restore patients’ audiovestibular function [22], such as intra-tympanical steroid administration [83]. However, since hyper-IgE syndrome is a genetic disease, the steroid treatment could only relieve the symptoms but not reverse the disease course.

Audiovestibular dysfunction related to hyper-IgE syndrome has a fluctuated course. This might be due to the reason that IgE memory could extend for several years and respond rapidly when exposured to allergens, so that IgE levels might be rapidly induced [73]. These fluctuating and suddenly increased IgE levels could explain the fluctuated and long-lasting course of recurrent sensorineural hearing loss [22]. Individuals with hyper-IgE syndrome may need long-term medical management (e.g., prophylactic antimicrobial regimens, immunomodulatory therapy on demand, and high-intensive personal hygiene). Without that, patients with hyper-IgE syndrome might encounter a significant burden related to the frequently recurrent infection and consequent audiovestibular dysfunction [5].

### 4.1. Clinical Recommendations

In addition to otolaryngological care, patients with hyper-IgE syndrome should also undergo neuro-otological and audiological assessments based on clinical recommendations. Although there has not been consensus regarding the red flag or timing for referral to an ENT doctor, we summarized some potential time points for referral consideration based on our previous report and clinical practice experience [12]. First, if patients, either with or without the documented genetic deficits of hyper-IgE syndrome, start to complain of audiological symptoms, such as decreased hearing function, tinnitus, and hyperacusis/hypoacusis. Second, if patients complain of unexplained vestibular symptoms, such as vertigo and dizziness. Third, clinicians should consider ENT doctor referral if patients’ audiovestibular symptoms are less likely related to ototoxicity of a currently prescribed medication. On the other hand, although subjective, clinicians should consider the option of ENT referral and pure-tone audiometry examination if his/her voice was frequently ignored by a specific patient. Similar with inner ear diseases related to other autoimmune diseases [9,10,11,12,13], the audiovestibular dysfunction related to hyper-IgE syndrome might be manageable if detected in a timely manner. However, delayed detection and management might lead to irreversible damage and consequent hearing loss and balance disorders.

### 4.2. Strengths and Limitations

This review’s strengths include adherence to PRISMA guidelines, comprehensive search strategies, and dual-author data extraction, providing a foundational synthesis despite limited evidence. However, the scarcity of direct hyper-IgE syndrome studies—relying on analogies from allergies—limits generalizability. The absence of histopathological data hinders mechanistic confirmation, and potential publication bias may overlook negative findings. Small sample sizes in case reports further constrain robustness.

### 4.3. Clinical Implications

Clinicians should maintain a high index of suspicion for audiovestibular symptoms in patients with hyper-IgE syndrome, prompting IgE testing and audiometric evaluation. Early referral to otolaryngologists for unexplained hearing loss or vertigo could facilitate timely immunomodulation, potentially averting progression [10,11,12,13]. Integrating allergy management into hyper-IgE syndrome care may mitigate risks, emphasizing multidisciplinary approaches. As mentioned above, the main method of management for audiovestibular dysfunction related to hyper-IgE syndrome was the prophylaxis in patients’ daily life. Specifically, personal hygiene to prevent fungal, bacterial, or viral infection was the cornerstone of hyper-IgE syndrome management. Further, temporarily prophylactic antibiotics or antifungal treatment might be necessary for frequently recurrent patients.

### 4.4. Future Directions

Prospective cohort studies are essential to establish hyper-IgE syndrome-specific prevalence and mechanisms, incorporating advanced imaging and genetic analyses. Randomized trials of biologics (e.g., dupilumab) could validate treatments, while exploring IgE’s role in vestibular hydrops may yield novel therapies. Longitudinal data on prognosis and quality of life impacts would inform guidelines, bridging immunology and otolaryngology. Additionally, we surely recognized the fact that numerous environmental factors were deeply involved in the pathogenesis of allergic reaction and IgE and/or histamine levels. Future genetic animal models studies regarding hyper-IgE syndrome should take the environmental factors into consideration at the same time [16].

## 5. Conclusion

This review article has synthesized the current knowledge about audiovestibular dysfunction related to hyper-IgE syndrome. We depicted a schematic diagram regarding the overall physiopathology of hyper-IgE syndrome associated with audiovestibular impairment in Figure 2. Audiovestibular dysfunction in hyper-IgE syndrome, driven by IgE-mediated inflammation, poses risks of sensorineural hearing loss and vestibular impairment. Routine audiometric and vestibular screening is critical to detect early symptoms, enabling timely immunomodulatory interventions to prevent irreversible disability and enhance quality of life. Multidisciplinary care integrating otolaryngology and immunology is essential. Future research should prioritize prospective studies to establish targeted therapies and optimize clinical outcomes for hyper-IgE syndrome patients.

## Data Availability

All the data of the current study are available at reasonable request to the corresponding authors.

## Supplementary Materials

The following supporting information can be downloaded at https://www.mdpi.com/article/10.3390/ijms26209932/s1. References [1,2,3,5,8,16,22,26,28,30,31,32,33,34,35,36,37,38,39,40,41,42,43,44,45,46,47,48,49,50,51,52,53,54,55,56,57,58,59,60,61,62,63,64,65,68,72,84,85,86,87] are cited in the supplementary materials.
