# Supplementary material for: Audiovestibular Dysfunction in Hyper-IgE Syndrome: A Systematic Review of Characteristics, Pathophysiology, Diagnosis, and Management
Source: Int J Mol Sci. 2025 Oct 12;26(20):9932. doi: 10.3390/ijms26209932 (PMC12564592; doi:10.3390/ijms26209932)
Supplement: Supplementary file 1 [file ijms-26-09932-s001.zip › ijms-3859421-supplementary.pdf]

Table S1: PRISMA 2020 checklist of current systematic review

Section and Topic	Item #	Checklist item	Page where item is reported
**TITLE**			
Title	1	Identify the report as a systematic review.	1
**ABSTRACT**			
Abstract	2	See the PRISMA 2020 for Abstracts checklist.	4
**INTRODUCTION**			
Rationale	3	Describe the rationale for the review in the context of existing knowledge.	6-7
Objectives	4	Provide an explicit statement of the objective(s) or question(s) the review addresses.	6-7
**METHODS**			
Eligibility criteria	5	Specify the inclusion and exclusion criteria for the review and how studies were grouped for the syntheses.	8-9
Information sources	6	Specify all databases, registers, websites, organisations, reference lists and other sources searched or consulted to identify studies. Specify the date when each source was last searched or consulted.	8-9
Search strategy	7	Present the full search strategies for all databases, registers and websites, including any filters and limits used.	8-9
Selection process	8	Specify the methods used to decide whether a study met the inclusion criteria of the review, including how many reviewers screened each record and each report retrieved, whether they worked independently, and if applicable, details of automation tools used in the process.	8-9
Data collection process	9	Specify the methods used to collect data from reports, including how many reviewers collected data from each report, whether they worked independently, any processes for obtaining or confirming data from study investigators, and if applicable, details of automation tools used in the process.	8-9
Data items	10a	List and define all outcomes for which data were sought. Specify whether all results that were compatible with each outcome domain in each study were sought (e.g. for all measures, time points, analyses), and if not, the methods used to decide which results to collect.	8-9
	10b	List and define all other variables for which data were sought (e.g. participant and intervention characteristics, funding sources). Describe any assumptions made about any missing or unclear information.	9-10
Study risk of bias assessment	11	Specify the methods used to assess risk of bias in the included studies, including details of the tool(s) used, how many reviewers assessed each study and whether they worked independently, and if applicable, details of automation tools used in the process.	9-10
Effect measures	12	Specify for each outcome the effect measure(s) (e.g. risk ratio, mean difference) used in the synthesis or presentation of results.	9-10
Synthesis methods	13a	Describe the processes used to decide which studies were eligible for each synthesis (e.g. tabulating the study intervention characteristics and comparing against the planned groups for each synthesis (item #5)).	Not done
	13b	Describe any methods required to prepare the data for presentation or synthesis, such as handling of missing summary statistics, or data conversions.	Not done

Section and Topic	Item #	Checklist item	Page where item is reported
	13c	Describe any methods used to tabulate or visually display results of individual studies and syntheses.	Not done
	13d	Describe any methods used to synthesize results and provide a rationale for the choice(s). If meta-analysis was performed, describe the model(s), method(s) to identify the presence and extent of statistical heterogeneity, and software package(s) used.	Not done
	13e	Describe any methods used to explore possible causes of heterogeneity among study results (e.g. subgroup analysis, meta-regression).	Not done
	13f	Describe any sensitivity analyses conducted to assess robustness of the synthesized results.	Not done
Reporting bias assessment	14	Describe any methods used to assess risk of bias due to missing results in a synthesis (arising from reporting biases).	9-10
Certainty assessment	15	Describe any methods used to assess certainty (or confidence) in the body of evidence for an outcome.	9-10
**RESULTS**			
Study selection	16a	Describe the results of the search and selection process, from the number of records identified in the search to the number of studies included in the review, ideally using a flow diagram.	11-12
	16b	Cite studies that might appear to meet the inclusion criteria, but which were excluded, and explain why they were excluded.	11-12
Study characteristics	17	Cite each included study and present its characteristics.	11-12
Risk of bias in studies	18	Present assessments of risk of bias for each included study.	11-12
Results of individual studies	19	For all outcomes, present, for each study: (a) summary statistics for each group (where appropriate) and (b) an effect estimate and its precision (e.g. confidence/credible interval), ideally using structured tables or plots.	11-12
Results of syntheses	20a	For each synthesis, briefly summarise the characteristics and risk of bias among contributing studies.	Not done
	20b	Present results of all statistical syntheses conducted. If meta-analysis was done, present for each the summary estimate and its precision (e.g. confidence/credible interval) and measures of statistical heterogeneity. If comparing groups, describe the direction of the effect.	Not done
	20c	Present results of all investigations of possible causes of heterogeneity among study results.	Not done
	20d	Present results of all sensitivity analyses conducted to assess the robustness of the synthesized results.	Not done
Reporting biases	21	Present assessments of risk of bias due to missing results (arising from reporting biases) for each synthesis assessed.	13-14
Certainty of evidence	22	Present assessments of certainty (or confidence) in the body of evidence for each outcome assessed.	13-14
**DISCUSSION**			
Discussion	23a	Provide a general interpretation of the results in the context of other evidence.	16-18
	23b	Discuss any limitations of the evidence included in the review.	16-18
	23c	Discuss any limitations of the review processes used.	16-18

Section and Topic	Item #	Checklist item	Page where item is reported
	23d	Discuss implications of the results for practice, policy, and future research.	19
**OTHER INFORMATION**			
Registration and protocol	24a	Provide registration information for the review, including register name and registration number, or state that the review was not registered.	5
	24b	Indicate where the review protocol can be accessed, or state that a protocol was not prepared.	5
	24c	Describe and explain any amendments to information provided at registration or in the protocol.	5
Support	25	Describe sources of financial or non-financial support for the review, and the role of the funders or sponsors in the review.	20
Competing interests	26	Declare any competing interests of review authors.	20
Availability of data, code and other materials	27	Report which of the following are publicly available and where they can be found: template data collection forms; data extracted from included studies; data used for all analyses; analytic code; any other materials used in the review.	20

The current checklist followed the latest PRISMA 2020 guideline.[1]

Table S2: Keyword and search results in each database

Database	Keyword	Filter	Date	Result
PubMed	(Job syndrome OR "Job's syndrome" OR Buckley syndrome OR Hyper-IgE syndrome) AND (hearing loss OR sensorineural hearing loss OR SNHL OR audiology OR tinnitus OR vertigo OR vestibular OR dizziness)	N/A	2025/08/06	23
Embase	(Job syndrome OR "Job's syndrome" OR Buckley syndrome OR Hyper-IgE syndrome) AND (hearing loss OR sensorineural hearing loss OR SNHL OR audiology OR tinnitus OR vertigo OR vestibular OR dizziness)	N/A	2025/08/06	0
ClinicalKey	(Job syndrome OR "Job's syndrome" OR Buckley syndrome OR Hyper-IgE syndrome) AND (hearing loss OR sensorineural hearing loss OR SNHL OR audiology OR tinnitus OR vertigo OR vestibular OR dizziness)	N/A	2025/08/06	336
Web of Science	(Job syndrome OR "Job's syndrome" OR Buckley syndrome OR Hyper-IgE syndrome) AND (hearing loss OR sensorineural hearing loss OR SNHL OR audiology OR tinnitus OR vertigo OR vestibular OR dizziness)	N/A	2025/08/06	23
ScienceDirect	(Job syndrome OR Buckley syndrome OR Hyper-IgE syndrome) AND (hearing loss OR audiology OR tinnitus OR vertigo OR vestibular OR dizziness)	N/A	2025/08/06	10621

Abbreviation: N/A: not applied

Table S3: Excluded studies and reason

Reason	Numbers	References
Animal study	1	[2]
Meta-analysis	1	[3]
Not related to audiovestibular dysfunction	3	[4-6]
Not related to hyper IgE syndrome	4	[7-10]
Review article not related to target disease	1	[11]

Table S4: Newcastle-Ottawa Scale and Characteristics for the Included Trial (observational trial)

Study	Selection				Comparability	Exposure			Total
	Case definition	Representative	Control selection	Control definition	Comparability	Ascertainment	Same method	Non-Response rate	Summary
Dave, T. (2024)[12]	*	*				*			3*
Esmaeilzadeh, M. (2024)[13]	*	*				*			3*
Sun, H. (2024)[14]	*	*	*	*	*	*	*		7*
Yaakoubi, R. (2023)[15]	*	*			*	*	*		**5***
Lachover-Roth, I. (2022)[16]	*	*	*	*	*	*	*		7*
Lan, J. (2022)[17]	*	*				*			3*
Ma, Y. (2021)[18]	*	*	*	*	*	*	*		7*
Awad, R. (2020)[19]	*	*				*			3*
Borst, J. (2020)[20]	*	*				*			3*
Singh, A. (2016)[21]	*	*				*			3*
Yanagimachi, M. (2016)[22]	*	*				*			3*
Patel, N.C. (2015)[23]	*	*				*			3*
Purkait, R. (2014)[24]	*	*				*			3*

Chandesris, M.O. (2012)[25]	*	*	*	*	*	*	*	7*
Goussetis, E. (2010)[26]	*	*				*		3*
Joshi, A.Y. (2009)[27]	*	*			*	*	*	5*
Sarmento, K.M. Jr. (2008)[28]	*	*				*		3*
Freeman, A.F. (2007)[29]	*	*			*	*	*	5*
Holland, S.M. (2007)[30]	*	*	*	*	*	*	*	7*
DeWitt, C.A. (2006)[31]	*	*				*		3*
Moin, M. (2006)[32]	*	*	*	*	*	*	*	7*
Kimura, A. (2005)[33]	*	*				*		3*
Renner, E.D. (2004)[34]	*	*			*	*	*	5*
Bilora, F. (2000)[35]	*	*				*		3*
Grimbacher, B. (1999)[36]	*	*			*	*	*	5*
Fernández, M. (1993)[37]	*	*				*		3*

* indicated this study have a good performance in this item

Table S5: Summary of the included study

Study	Characteristics		Outcomes	Summary
	Study design	Recruited case characteristics	Results	Conclusion
AlYafie, R. (2025)[38]	Review article	Not applicable	Hyper IgE syndromes (HIES) form a rare group of primary immunodeficiency disorders (PIDs) distinguished by persistent skin abscesses, dermatitis, allergies, and infections, in addition to their characteristic high serum IgE levels. Autosomal dominant (AD) and autosomal recessive (AR) genetic defects have been reported in HIES. From a clinical perspective, AD-HIES cases generally exhibit several non-immunologic features, including connective tissue, dental and skeletal abnormalities, whilst AR-HIES conditions have a higher incidence of neurologic complications and cutaneous viral infections	As a result, HIES patients are predisposed to recurrent bacterial and/or fungal infections, as well as atopic allergic responses. In many cases, the exact biological mechanisms responsible for the variations observed in the clinical phenotypes between the two inherited forms of HIES are still unclear. In this review, we describe the genetic basis of HIES with a distinction between the AR-HIES and AD-HIES forms, to better comprehend the different underlying molecular mechanisms, a distinction which is imperative for the accurate diagnosis, management, and development of targeted therapies for HIES patients
Salehi, M. (2025)[39]	Review article	Not applicable	Hyper IgE syndromes (HIES) are rare primary immunodeficiency characterized by susceptibility to specific infections, eczema, and elevated IgE levels. Pathogenic mutations in *STAT3*, *IL6R*, *IL6ST*, *ERBB2IP*, *PGM3*, *ZNF431, SPINK5, TGFBR1/2*, and *CARD11* have been identified as genetic factors contributing to phenotypes of HIES lead to hindered differentiation and activity, aberrant signaling cascades and disrupting immune regulation	Although rare, HIES significantly impacts patients due to its complex medical manifestations and need for lifelong management. Identifying casual variants is essential for effective clinical management of these complex conditions.

Sutanto, H. (2025)[40]	Review article	Not applicable	Recent research has identified several pivotal genetic mutations, including those in STAT3, DOCK8, and PGM3, which play critical roles in disrupting immune pathways such as Th17 differentiation and IgE regulation. These molecular defects have been linked to the hallmark features of hyper IgE syndrome, including recurrent infections and elevated serum IgE levels, as well as its overlap with atopic conditions like eczema, asthma, and food allergies.	This review highlights the role of specific genetic mutations in shaping the clinical and immunological phenotype of HIES. Key takeaways include the necessity of integrating molecular insights with clinical observations for accurate diagnosis and the potential of emerging targeted therapies to address both immunological and allergic aspects of the syndrome.
Dave, T. (2024)[12]	Case report	A 37-year-old male with hyper IgE syndrome, diagnosed at the age of 2 due to recurrent cold abscesses caused by Staphylococcal infections..	The patient exhibited typical symptoms, including recurrent eczema, frequent bacterial infections, mucocutaneous candidiasis, and various physical abnormalities. Diagnostic markers such as elevated IgE levels and eosinophilia supported the HIES diagnosis, which was further confirmed by the identification of a STAT3 gene mutation. Treatment primarily involved supportive measures and antibiotics for infections.	This case report highlights the importance of early diagnosis, prompt management of infections, and the need for ongoing research to improve our understanding and treatment of hyper IgE syndrome.
Esmaeilzadeh, M. (2024)[13]	Case report	A 5-year-old girl with elevated serum IgE levels had tooth decay and multiple dental abscesses with multiple eczemas, and she was receiving treatment with warfarin due to a history of jugular vein thrombosis. Angular cheilitis, de-papillation of the tongue, deep furrows on the tongue, numerous intraoral ulcerated lesions, poor oral hygiene, and gingivitis were seen in the intraoral examination.	Hyper IgE syndrome is diagnosed before the patients come to the dentist for their dental treatment; the role of a specialized dentist, especially a pedodontist, is paramount. The prognosis for such patients dramatically depends on the early detection and effective management of its complications. By recognizing and managing these oral symptoms promptly, pedodontists can significantly improve the quality of life and overall health outcomes for such patients.	Dentists play an essential role in the early diagnosis of HIES and in monitoring their oral health conditions. Timely extraction of over-retained primary teeth can reduce the necessity for complex treatments, thereby facilitating the management of patients with Job's syndrome.

Sun, H. (2024)[14]	Cohort	Used RT-PCR to compare the expression of AMPs and a fungistasis assay to evaluate the antifungal activity of sinus secretions. Using flow cytometry, we characterized the presence of T_H17/T_H22 cells and signal transducer and activator of transcription (STAT) signaling from AFRS patients, non-AFRS CRSwNP patients, and healthy controls	Decreased expression of AMPs in AFRS sinus mucosa with concordant decrease in antifungal activity in sinus secretions was noticed. IL-22 and IL-22-producing T cells were deficient within sinus mucosa of AFRS patients. In vitro studies demonstrated a defect in IL-6/STAT3 signaling critical for T_H17/T_H22 differentiation. Epithelial cells from AFRS patients could express AMPs when stimulated with exogenous IL-22/IL-17 and circulating T_H17 cell abundance was normal	Similar to other hyper-IgE syndromes, but distinct from CRSwNP, AFRS patients express a defect in STAT3 activation limited to IL-6-dependent STAT3 phosphorylation that is critical for T_H17/T_H22 differentiation. This defect leads to a local deficiency of IL-17/IL-22 cytokines and deficient AMP expression within diseased sinus mucosa of AFRS patients.
Yaakoubi, R. (2023)[15]	Cohort	Nine Tunisian and two Libyan patients with clinical suspicion of Hyper IgE syndrome were investigated at Pasteur Institute of Tunis.	The present study highlights the diagnostic challenge in eleven patients with the clinical phenotype of HIES in a resource-limited region. Candidate gene strategy supported by clinical features, laboratory findings and functional investigations allowed the identification of two heterozygous STAT3 mutations in five patients, and a bi-allelic DOCK8 mutation in one patient. Whole Exome Sequencing allowed to unmask atypical presentations of DOCK8 deficiency in two patients presenting with clinical features reminiscent of STAT3 deficiency.	Our study underlies the importance of the differential diagnosis between STAT3 and DOCK8 deficiencies in order to improve diagnostic criteria and to propose appropriate therapeutic approaches. In addition, our findings emphasize the role of NGS in detecting mutations that induce overlapping phenotypes.
Gharehzadehshirazi, A. (2022)[41]	Review article	Not applicable	Hyper IgE syndromes (HIESs) are a group of rare inborn errors of immunity with a triad of eczema, increase susceptibility to sinopulmonary and skin infections with high level of IgE serum.	There is yet no specific curative treatment for most of HIESs at present, and the treatments are mostly standing on early diagnosis and preventive therapies. For instance, the genetic diagnosis is an important module, while, due to DOCK8 mutations, the hematopoietic stem cell transplantation is necessary

				for patients with autosomal recessive form of HIESs
Lachover-Roth, I. (2022)[16]	Case-control	Three hundred DNA samples from 300 females, were obtained from the medical genetics institute in Meir Medical center. The DNA samples were divided into three groups: A. 100 samples from the studied village whose surname was the same as the surname of the patients. B. 100 samples from the studied village with a different surname. C. 100 samples of Muslim females from other villages, in geographic vicinity. Those samples served as a control group. It was taken into consideration that groups A & B can be mixed due to changing family name after marriage.	Heterozygous nonsense mutation in ZNF341 was found in ten samples (5%) of the study group compared to zero in the control group ($p<0.01$).	The carrier frequency of the mutation in ZNF341 in the studied village population is 1:20. This high frequency is probably due to founder mutation and consanguineous marriages.
Lan, J. (2022)[17]	Case report	A 28-year-old Chinese woman was admitted for recurrent cough for 7 years, markedly elevated serum IgE level, and recurrent pneumonia caused by multiple pathogens	Using whole-exome sequencing, the *STAT3* (c.1294G>T, p.Val432Leu) missense mutation for the autosomal dominant hyper-IgE syndrome was identified, and omalizumab was prescribed at 300 mg every 2 weeks. The patient responded well with the improvement of respiratory symptoms and lung function tests. The level of serum IgE remained stable on follow-up	Omalizumab treatment proved beneficial in the case of HIES, especially with chronic airway disease, for which therapeutic options are limited. However, larger-scale prospective studies and long-term follow-up are required to establish the efficacy and safety of this therapeutic intervention
Ma, Y. (2021)[18]	Case-control	A total of 242 subjects with sudden onset hearing loss, including 115 with acute low-tone sensorinerual hearing loss (ALHL) and 127 with idiopathic sudden sensorineural hearing loss (ISSHL), were included in this study.	Compared to the values in the ISSHL group, a significantly younger onset age, higher female onset proportion, increased total IgE level and specific IgE level were noted in the ALHL group. Total IgE and specific IgE levels were factors that contributed to the SP/AP ratio in the	High IgE levels correlated with an enhanced SP/AP ratio in ALHL. High IgE levels could be used as a predictor of ALHL recurrence and MD transformation.

			electrocochleogram in ALHL group. Finally, during the follow-up for the ALHL group, 37 subjects recurred, and 17 subjects developed Meniere Disease.	
Minegishi, Y. (2021)[42]	Review article	Not applicable	The original hyper-IgE syndrome is characterized by diminished inflammatory response, in combination with Staphylococcus aureus skin abscess and pneumonia followed by pneumatocele formation. These immunological manifestations are frequently associated with skeletal and connective tissue abnormalities.	The fact that the non-immune manifestations of the gain-of-function mutations of TGFBR1 and TGFBR2 are similar to the those of dominant negative mutations of STAT3 provide a clue to elucidate molecular mechanisms of non-immune manifestations of hyper-IgE syndrome. Research on this hereditary atopic syndrome is being actively conducted to elucidate the molecular mechanisms and to develop new therapeutic approaches
Tsilifis, C. (2021)[43]	Review article	Not applicable	The hyper-IgE syndromes (HIES) are a heterogeneous group of inborn errors of immunity sharing manifestations including increased infection susceptibility, eczema, and raised serum IgE. Since the prototypical HIES description 55 years ago, areas of significant progress have included description of key disease-causing genes and differentiation into clinically distinct entities. The first two patients reported had what is now understood to be HIES from dominant-negative mutations in signal transduction and activator of transcription 3 (STAT3-HIES), conferring a broad immune defect across both innate and acquired arms, as well as defects in skeletal, connective tissue, and vascular function,	Research into the pathophysiology of STAT3-HIES has driven understanding of the interface of several signaling pathways, including the JAK-STAT pathways, interleukins 6 and 17, and the role of Th17 lymphocytes, and has been expanded by identification of phenocopies such as mutations in IL6ST and ZNF341. In this review we summarize the published literature on STAT3-HIES, present the diverse clinical manifestations of this syndrome with current management strategies, and update on the uncertain role of stem cell transplantation for this disease. We

			causing a clinical phenotype including eczema, staphylococcal and fungal skin and pulmonary infection, scoliosis and minimal trauma fractures, and vascular tortuosity and aneurysm	outline key unanswered questions for further study
Awad, R. (2020)[19]	Case report	A 17-year-old male with typical feature of hyper IgE syndrome with atopic dermatitis and frequent infection.	Hyper IgE syndrome is a medical condition that can be sporadic or hereditary. It consists of multiple overlapping primary immunodeficiency conditions and is characterized by a classical triad of high immunoglobulin E levels, recurrent pneumonia with pneumatocele and recurrent cold skin abscesses from staphylococcus infections. Eosinophilia is also common in HIES patients. HIES is often underdiagnosed in Syria as it cannot be confirmed without genetic testing, which is unavailable across Syria for HIES.	Other cases in a regional country carried atypical novel mutations, which may indicate that these mutations may exist in Syria as well. However, our case had findings that were not reported with other HIES cases. Determining these genes in the case presented was not possible, and future studies need to overcome this hurdle.
Borst, J. (2020)[20]	Case report	A 23-year-old woman with autosomal dominant hyper-IgE syndrome complicated by recurrent pneumonia and sinusitis presented with 1 week of multiple painful oral ulcers unresponsive to empiric antiviral and antifungal treatment.	Biopsy revealed chronic active inflammation with no evidence of viral inclusion bodies or fungal hyphae. She was diagnosed with recurrent aphthous stomatitis and referred to a local dentist for CO_2 laser treatments with rapid resolution of her symptoms.	This case highlights the broad differential for recurrent oral ulcers in people with a primary immunodeficiency. It also raises awareness of the benefits of laser therapy for aphthous stomatitis treatment and the importance of partnering with our colleagues in dentistry.
Tavakol, M. (2019)[44]	Review article	Not applicable	Hyper IgE syndromes are classified as groups of primary immunodeficiency diseases, which are presented with a series of symptoms including recurrent infections accompanied by elevated serum IgE level and some atopic features. Both autosomal dominant and recessive mutations may lead to hyper IgE syndrome.	Connective tissue, skeletal and vascular abnormalities are prominent in autosomal dominant form, while in autosomal recessive form, viral infections, malignancies and neurological disorders are more

				prominent. The definite diagnosis is made by mutation analysis.
Devilliers, H. (2018)[45]	Review article	Not applicable	Hyper-IgE may be found under many pathological conditions. The role of IgE is essentially associated with the occurrence of allergic manifestations, which may be accompanied by an increase of their serum levels.	Elevation of total IgE has also been reported in association with certain rare genetic immune deficiencies called hyper-IgE syndromes. Other circumstances such as infectious diseases, tumors or autoimmune diseases may also be accompanied by an excessive synthesis of IgE.
Ponsford, M. J. (2018)[46]	Review article	Not applicable	Interface between allergy and immunodeficiency, highlighting key clinical scenarios which should draw attention to possible immunodeficiency associated with extreme elevation of IgE, and outline initial laboratory assessment and management.	Through this review, the authors summarized the characteristics of hyper IgE syndrome, including its neonatal complication, infantile eczema/infection, connective disorder complication, autoimmunity issue, and related treatment strategy.
Hashemi, H. (2017)[47]	Review article	Not applicable	The hyperimmunoglobulin E syndromes (HIESs) are very rare immunodeficiency syndromes with multisystem involvement, including immune system, skeleton, connective tissue, and dentition. HIES are characterized by the classic triad of high serum levels of immunoglobulin E (IgE), recurrent staphylococcal cold skin abscess, and recurrent pneumonia with pneumatocele formation	The diagnosis of HIES is inconclusive, dependent on the evolution of a constellation of complex multisystemic symptoms and signs which develop over the years. Until time, no treatment modality is curative for basic defect in HIES, in terms of cytokines/chemokines derangement
Singh, A. (2016)[21]	Case report	A symptomatic 6-year-old boy with typical feature of hyper IgE syndrome	Since his early infancy with recurrent respiratory problems, rash, organomegaly, suppurative generalised lymphadenopathy, he was misdiagnosed as tuberculosis twice. Following extensive investigations, infectious causes for such manifestations were ruled out and the final diagnosis of possible hyper IgE	The diagnosis of hyper IgE syndrome relies on a combination of clinical features and laboratory studies. Heterogeneous manifestations of mimics common infections prevalent in tropical areas and results in delayed diagnosis. Primary goal of

			syndrome was made as genetic studies could not be done.	treatment lies in prevention of infections with prophylactic antibiotics.
Yanagimachi, M. (2016)[22]	Case report	Followed for more than 8 years two patients with AD-HIES who were treated with HSCT. Their ability of IL-17 production was evaluated by flow cytometry	Both patients indicated the normal ability of IL-17 production and their serum IgE levels decreased after HSCT. On the other hand, they suffered from pulmonary complications of AD-HIES such as pneumatoceles and bronchiectasis even after HSCT; however, the frequency of infections was decreased	Although the dysfunction of STAT3 in non-hematological tissues such as the lungs could not be corrected by HSCT, AD-HIES patients with risk factors for pulmonary complications may benefit from immunological correction by HSCT before severe pulmonary complications occur. Future studies should investigate risk factors for pulmonary complications in AD-HIES patients
Patel, N.C. (2015)[23]	Case report	A 14-year old female with AD-HIES developed recurrent methicillin-resistant Staphylococcus aureus (MRSA) abscesses. Immunologic analysis showed elevated IgE (4331 kU/L), absent Th17 cells, and markedly decreased STAT3 phosphorylation in cytokine stimulated peripheral blood mononuclear cells.	She underwent T-cell depleted haploidentical HSCT from her father following reduced intensity conditioning. She developed one MRSA hand abscess after transplant. Twenty-four months post transplant, she had complete donor chimerism (>95 % donor), normal absolute T cell numbers, and a normal percentage of Th17 cells. IgE was normal at 25 kU/L. She remains well 42 months after transplantation off all antibacterial prophylaxis	Haploidentical HSCT led to successful bone marrow engraftment, normalization of STAT3 signaling in hematopoietic cells, normalization of IgE, and restoration of immune function in this patient with AD-HIES
Purkait, R. (2014)[24]	Case report	A 5-year-old male child with hyper- IgE syndrome who presented with neurologic manifestations of acute disseminated encephalomyelitis	The hyper-immunoglobulin E (IgE) syndrome (HIES), also known as Job's syndrome is a rare primary immunodeficiency characterized by the clinical triad of recurrent staphylococcal abscesses of skin, recurrent cyst-forming pneumonia, and an elevated serum IgE level of > 2000 IU/ml. Although, most cases are sporadic, families with autosomal dominant	Very few articles were published previously on central nervous system abnormalities with definite neurologic manifestations which may vary from partial facial nerve paralysis to hemiplegia in children but Acute Disseminated Encephalomyelitis (ADEM) in a child with HIES hitherto has not been reported.

			(AD-HIES) and recessive (AR-HIES) traits have been reported.	
Chandesris, M.O. (2012)[25]	Case-control	Genomic DNA was prepared from the blood samples of patients and controls by the standard phenol-chloroform extraction method	Autosomal dominant deficiency of signal transducer and activator of transcription 3 (STAT3) is the main genetic etiology of hyper-immunoglobulin (Ig) E syndrome. We documented the molecular, cellular, and clinical features of 60 patients with heterozygous STAT3 mutations from 47 kindreds followed in France. We identified 11 known and 13 new mutations of STAT3	Overall, the prognosis of STAT3 deficiency may be considered good, provided that multiple prophylactic measures, including IgG infusions, are implemented
Goussetis, E. (2010)[26]	Case report	The cases of 2 unrelated boys with sporadic HIES complicated by high-grade non-Hodgkin lymphoma, who were cured with myeloablative HLA-matched sibling bone marrow transplantation. Before presenting for HSCT, both patients had experienced recurrent infections despite receiving antibiotic prophylaxis. Non-Hodgkin lymphoma was treated by 7 and 4 CHOP (Cyclophosphamide Hydroxydaunorubicin Oncovin Prednisone) chemotherapy cycles in patients 1 and 2, respectively, achieving complete remission in patient 1 only	Both patients underwent allogeneic bone marrow stem cell transplantation after written informed consent, each from their respective healthy HLA-identical sibling. Neither patient had acute or chronic GVHD. Complete donor-derived hematopoietic chimerism has consistently been detected after day +60 after transplantation by studying short tandem repeats. Full donor chimerism was also detected on purified $CD3^+$ cells at both patients' most recent follow-up visit. IgE levels were normal (<250 IU/mL) 2 months after transplantation for the first time and remain normal 14 and 10 years after transplantation for patients 1 and 2, respectively.	No further transplantation attempts in patients with HIES have been reported. The clinical course of the patients presented here demonstrates that manifestations of AD-HIES related to the STAT-3 defect in lymphohematopoietic cells can be resolved by myeloablative allogeneic HSCT. The length of follow-up enables us to suggest that the progression of other significant deficits associated with AD-HIES, including neurological, musculoskeletal, and vascular abnormalities, might also be delayed or halted. Allogeneic HSCT for patients with AD-HIES presenting progressive lung disease or lymphoproliferative disease should be considered, despite the possibility of long-term complications, such as GVHD, especially if an HLA-identical sibling is available

Milner, (2010)[48]	J.D.	Review article	Not applicable	Patients with mutations in STAT3, who lack Th17 cells, develop AD-HIES, whereas other disorders of elevated IgE may be caused by mutations in Tyk2 or DOCK8, the latter of which is associated with decreased expansion of CD8 more so than CD4 T cells. Recent studies on patients with recurrent mucocutaneous candidiasis have led to the discovery of mutations in CARD9 and DECTIN-1, genes key to the production of the Th17-driving cytokines IL-1beta, IL-6, and IL-23.	Studies of the peripheral blood of HIV-positive patients have shown a decreased Th17:Th1 ratio, and Th17 cells were preferentially depleted from the gastrointestinal tract within weeks of simian immunodeficiency virus infection in rhesus macaques.
Freeman, (2009)[49]	A.F.	Review article	Not applicable	Autosomal dominant Hyper IgE syndrome (AD-HIES) is a rare primary immunodeficiency characterized by eczema, recurrent skin and lung infections, elevated serum IgE, and various connective tissue, skeletal, and vascular abnormalities. Mutations in Signal transducer and activator of transcription 3 (STAT3) have recently been found to account for the majority of cases, however the pathogenesis of the varied features remains poorly defined	The hyperintensities seen on brain MRI in HIES may relate to increased inflammation, demyelination, and astrocytosis after neurologic injury, as seen in astrocyte-specific *Stat3* knockout mice
Joshi, (2009)[27]	A.Y.	Cohort	Identified case of hyper IgE syndrome through database research	Elevated serum immunoglobulin E can be caused by allergies, infections and immune conditions including hyper IgE syndrome, which is a rare primary immunodeficiency disease most commonly characterized by a triad of findings, including increased serum IgE levels, recurrent skin abscesses, and pneumonias leading to pneumatocele formation.	There was a statistically significant association between IgE levels and the severity of eczema (p = 0.009). Ninety percent of the subjects with IgE level >or=2000 IU/mL did not have HIES. There was no correlation between IgE levels and the diagnosis of HIES (p = 0.5). A variety of clinical situations result in an elevated IgE level, with atopy being the most common cause. In the absence of typical clinical features, elevated

				serum IgE levels are not predictive of HIES
Freeman, A.F. (2008)[50]	Review article	Not applicable	The hyper IgE syndromes (HIES) are rare primary immune deficiencies characterized by elevated serum IgE, rash and recurrent bacterial infections of the skin and lung. Autosomal dominant HIES, the most common disease in this group, results from STAT3 mutations and has a variety of connective tissue and skeletal abnormalities. The genetic etiologies of the more rare autosomal recessive form(s) still need delineation.	The etiology of the neurologic complications was not clear for all patients, but one had a cerebral cryptococcoma with meningitis, while others had severe central nervous system vasculitis. Treatment of these syndromes has relied on prophylactic and therapeutic antimicrobials and aggressive skin care.
Sarmento, K.M. Jr. (2008)[28]	Case report	A 14-year-old girl of Dubowitz syndrome, hyper-IgE syndrome, and nasal polyposis (due to allergic fungal sinusitis).	Eosinophilic inflammatory reaction is the feature present in all three conditions. Unlike most cases of allergic fungal sinusitis, this case was not treated with an initial booster of oral steroids due to the risk of disseminated invasive fungal infection, reported in other cases of hyper-IgE syndrome.	The hyper-immunoglobulin E (hyper-IgE or Job syndrome) is a primary immunodeficiency characterized by recurrent staphylococcal abscesses, recurrent cyst-forming pneumonia, and an elevated serum IgE level of >2000 IU/ml.
Freeman, A.F. (2007)[29]	Retrospective cohort	The authors reviewed the medical records and autopsy slides of 6 patients with HIES with autopsies performed at our institution	All 6 patients with HIES were women and ranged in age from 24 to 40 years. All patients had a history of cystic lung disease and had pneumonia at the time of death, with Pseudomonas aeruginosa and fungal organisms predominating. Pulmonary fungal vascular invasion with fatal hemorrhage was observed in 3 patients, and metastatic fungal disease to the brain was observed in 2 patients caused by Aspergillus fumigatus and Scedosporium prolificans. Four patients had evidence of renal tubular injury, which was likely from amphotericin B toxicity; 3 patients had	This finding highlights the important role Pseudomonas and Aspergillus species play in patients with HIES with cystic lung disease. Intensified antifungal and gram-negative bacterial prophylaxis need evaluation as possible strategies to prevent these infectious complications in patients with cystic lung disease

			glomerulosclerosis; and 1 patient had 2 kidney angiomyolipomas	
Holland, S.M. (2007)[30]	Case-control	Longitudinal clinical data on patients with the hyper-IgE syndrome and their families and assayed the levels of cytokines secreted by stimulated leukocytes and the gene expression in resting and stimulated cells	The authors found increased levels of proinflammatory gene transcripts in unstimulated peripheral-blood neutrophils and mononuclear cells from patients with the hyper-IgE syndrome, as compared with levels in control cells. In vitro cultures of mononuclear cells from patients that were stimulated with lipopolysaccharide, with or without interferon-gamma, had higher tumor necrosis factor alpha levels than did identically treated cells from unaffected persons (P=0.003). In contrast, the cells from patients with the hyper-IgE syndrome generated lower levels of monocyte chemoattractant protein 1 in response to the presence of interleukin-6 (P=0.03), suggesting a defect in interleukin-6 signaling through its downstream mediators, one of which is STAT3. We identified missense mutations and single-codon in-frame deletions in STAT3 in 50 familial and sporadic cases of the hyper-IgE syndrome. Eighteen discrete mutations, five of which were hot spots, were predicted to directly affect the DNA-binding and SRC homology 2 (SH2) domains	Mutations in STAT3 underlie sporadic and dominant forms of the hyper-IgE syndrome, an immunodeficiency syndrome involving increased innate immune response, recurrent infections, and complex somatic features
DeWitt, C.A. (2006)[31]	Case report	Patient 1 was an 18-month-old African American boy with recurrent staphylococcal cold abscesses, pneumonia, and bacteremia. Patient 2 was a 15-year-old Caucasian boy with long-standing hyperimmunoglobulin E syndrome.	Hyperimmunoglobulin E syndrome (HIES) is a rare immunodeficiency associated with elevated serum IgE levels, eczematous skin, recurrent cutaneous infections, and distinctive musculoskeletal features.	Occasional episodes of cold abscesses and sinusitis, but has had excellent control since institution of this regimen and has not experienced any adverse effects

Moin, M. (2006)[32]	Case-control	The medical records of 22 patients from 21 unrelated families, who had been registered in the Iranian Primary Immunodeficiency Registry, were observed	IgE level was higher than 2000 IU/ml in all patients, ranging from >2000 to 80,000 IU/ml. The most commonly occurring manifestations were: eczema and dermatitis, pneumonia, upper respiratory tract infections, cutaneous abscesses, diarrhoea, deep abscesses, and otitis media. Other less frequent manifestations were: mucocutaneous candidiasis, sinusitis, cutaneous ulcers, Molluscum contagiosum, herpetic keratitis, onychomycosis, conjunctivitis, septic arthritis, and meningitis.	The hyper-immunoglobulin E syndrome is a multisystem disorder that affects especially cutaneous, respiratory, skeletal and the immune system. Although HIES is a rare condition, the recurrent infections should always raise a suspicion, which deserves further evaluation for detecting the syndrome
Kimura, A. (2005)[33]	Case report	A case of a 46 year old man with chronic inflammatory demyelinating polyneuropathy with hyper IgE. The patient presented with bilateral weakness, generalized hyporeflexia, and mild paresthesia of the fingers of both hands	Nerve conduction studies revealed multiple sites of motor conduction block in the absence of sensory abnormalities. Muscle strength increased, as did compound muscle action potential (CMAP) amplitude immediately after the intravenous infusion of immunoglobulin (IVIg).	Serum IgE levels also fluctuated in parallel with his relapsing-remitting clinical course. We propose that pure motor CIDP may be immune mediated and suggest that IgE-mediated allergy may be one potential cause of this condition
Renner, E.D. (2004)[34]	Case series	13 human immunodeficiency virus-seronegative patients from six consanguineous families with an autosomal-recessive form of hyperimmunoglobulin E syndrome (AR-HIES) and 68 of their relatives	Central nervous system sequelae, including hemiplegia, ischemic infarction, and subarachnoid hemorrhages, were common and contributed to high mortality. Notably, patients with AR-HIES did not have skeletal or dental abnormalities and did not develop pneumatoceles, as seen in AD-HIES	The autosomal-recessive form of hyperimmunoglobulin E syndrome is a primary immunodeficiency with elevated immunoglobulin E, eosinophilia, vasculitis, autoimmunity, central nervous system symptoms, and high mortality. AR-HIES lacks several of the key findings of AD-HIES and therefore represents a different, previously unrecognized disease entity
Bilora, F. (2000)[35]	Case report	A 37-year-old woman with hyper IgE syndrome and pneumonia	Job's syndrome (or hyperimmunoglobulinemia E syndrome) is a rare genetic disease characterized by skin eczema, pyogenic "cold"	We measured IgE serum by immuno-fluorometric test and neutrophil chemotaxis by migration in a Boyden

			abscesses, sinopulmonary recidivous infections and high IgE plasma concentrations. Job's syndrome treatment is not satisfactory and cases studied are still limited.	chamber before and after IVIG therapy. A moderate dose of IVIG resolved the clinical-radiological signs of the S. aureus bronchopneumonia and improved cytologic and biohumoral parameters. Intravenous immunoglobulins represent a useful treatment for acute pneumonia in Job's syndrome.
Grimbacher, B. (1999)[36]	Cohort	Nineteen kindreds with multiple cases of hyper IgE syndrome were scored for clinical and laboratory findings and were genotyped with polymorphic markers in a candidate region on human chromosome 4	The hyper-IgE syndrome is a rare primary immunodeficiency characterized by recurrent skin abscesses, pneumonia, and highly elevated levels of serum IgE. Hyper IgE syndrome is now recognized as a multisystem disorder, with nonimmunologic abnormalities of the dentition, bones, and connective tissue. HIES can be transmitted as an autosomal dominant trait with variable expressivity.	Linkage analysis showed a maximum two-point LOD score of 3.61 at recombination fraction of 0 with marker D4S428. Multipoint analysis and simulation testing confirmed that the proximal 4q region contains a disease locus for hyper IgE syndrome.
Fernández, M. (1993)[37]	Case report	An 11-year-boy with no relevant personal or family history demonstrated symptoms of hyper IgE syndrome.	Hyper-IgE syndrome is basically characterized by recurrent infections, chronic eczematous lesions, specific IgE antibodies against Staphylococcus aureus and markedly high serum IgE values.	Complementary studies revealed peripheral eosinophilia, increase in globular sedimentation rate and IgE values of 20,000 IU/ml, a nonspecific reaction to skin tests, and a skin biopsy compatible with atopic dermatitis. The differential diagnoses are discussed, as well as new diagnostic-therapeutic possibilities
Hsu, A.P. (1993)[51]	Review article	Not applicable	*STAT3* hyper IgE syndrome (*STAT3*-HIES) is a primary immune deficiency syndrome characterized by elevated serum IgE, eczema, and recurrent skin and respiratory tract infections, together with several nonimmune features. This disorder typically manifests in the	Survival is typically into adulthood, with most individuals now living into or past the sixth decade. Most deaths are associated with gram-negative (*Pseudomonas*) or filamentous fungal pneumonias resulting in hemoptysis.

		newborn period with a rash (often diagnosed as eosinophilic pustulosis) that subsequently evolves into an eczematoid dermatitis. Recurrent staphylococcal skin boils and bacterial pneumonias usually manifest in the first years of life	Lymphomas occur at an increased frequency.

Reference list of supplement tables:

1. Page, M.J.; McKenzie, J.E.; Bossuyt, P.M.; Boutron, I.; Hoffmann, T.C.; Mulrow, C.D.; Shamseer, L.; Tetzlaff, J.M.; Akl, E.A.; Brennan, S.E.; et al. The PRISMA 2020 statement: an updated guideline for reporting systematic reviews. *Bmj* **2021**, *372*, n71, doi:10.1136/bmj.n71.
2. Okada, S.; Nakamura, M.; Katoh, H.; Miyao, T.; Shimazaki, T.; Ishii, K.; Yamane, J.; Yoshimura, A.; Iwamoto, Y.; Toyama, Y.; et al. Conditional ablation of Stat3 or Socs3 discloses a dual role for reactive astrocytes after spinal cord injury. *Nat Med* **2006**, *12*, 829-834, doi:10.1038/nm1425.
3. Klokman, V.W.; Koningstein, F.N.; Dors, J.W.W.; Sanders, M.S.; Koning, S.W.; de Kleijn, D.P.V.; Jie, K.E. Blood biomarkers for the differentiation between central and peripheral vertigo in the emergency department: a systematic review and meta-analysis. *Acad Emerg Med* **2024**, *31*, 371-385, doi:10.1111/acem.14864.
4. Zhao, Q.; Sun, Y.; Sun, Y.; Liu, H.; Zhang, F. Upadacitinib for Treatment of Hyper-IgE Syndrome. *JAMA Dermatol* **2025**, doi:10.1001/jamadermatol.2025.2699.
5. Buckley, R.H.; Wray, B.B.; Belmaker, E.Z. Extreme hyperimmunoglobulinemia E and undue susceptibility to infection. *Pediatrics* **1972**, *49*, 59-70.
6. Davis, S.D.; Schaller, J.; Wedgwood, R.J. Job's Syndrome. Recurrent, "cold", staphylococcal abscesses. *Lancet* **1966**, *1*, 1013-1015, doi:10.1016/s0140-6736(66)90119-x.
7. Thorsberger, M.; Porsbjerg, C.; Yde, J.; Aanæs, K. Effects on hearing and tinnitus following Dupilumab treatment of severe asthma with chronic rhinosinusitis - a case report. *Rhinology Online* **2021**, *4*, 73 - 76, doi:10.4193/RHINOL/21.002.
8. Zhang, N.; Lyu, Y.; Guo, J.; Liu, J.; Song, Y.; Fan, Z.; Li, X.; Li, N.; Zhang, D.; Wang, H. Bidirectional Transport of IgE by CD23 in the Inner Ear of Patients with Meniere's Disease. *Journal of immunology (Baltimore, Md. : 1950)* **2022**, *208*, 827-838, doi:10.4049/jimmunol.2100745.
9. Jung, H.J.; Yu, G.H.; Park, J.H. Hypereosinophilic syndrome presenting with bilateral ear fullness. *Eur Ann Otorhinolaryngol Head Neck Dis* **2019**, *136*, 199-201, doi:10.1016/j.anorl.2018.03.009.
10. Lasisi, A.O.; Abdullahi, M. The inner ear in patients with nasal allergy. *J Natl Med Assoc* **2008**, *100*, 903-905, doi:10.1016/s0027-

9684(15)31403-6.
11. Zeng, B.; Domarecka, E.; Kong, L.; Olze, H.; Scheffel, J.; Monino-Romero, S.; Siebenhaar, F.; Szczepek, A.J. A systematic review of the clinical evidence for an association between type I hypersensitivity and inner ear disorders. *Front Neurol* **2024**, *15*, 1378276, doi:10.3389/fneur.2024.1378276.
12. Dave, T.; Tashrifwala, F.A.A.; Rangwala, U.S.; Hameed, R. Hyper-IgE syndrome: a case report. *Ann Med Surg (Lond)* **2024**, *86*, 1205-1209, doi:10.1097/MS9.0000000000001670.
13. Esmaeilzadeh, M.; Soleymani, A.A.; Mozafar, S.; Tariverdi, N.; Fatemi, S.A.; Khosrozamiri, M. Hyper IgE (Job's) Syndrome: A Primary Immune Deficiency with Oral Manifestations. *J Dent Sch* **2024**, *42*, 180-183, doi:10.22037/jds.v42i4.45398.
14. Sun, H.; Knight, J.M.; Li, Y.D.; Ashoori, F.; Citardi, M.J.; Yao, W.C.; Corry, D.B.; Luong, A.U. Allergic fungal rhinosinusitis linked to other hyper-IgE syndromes through defective T(H)17 responses. *J Allergy Clin Immunol* **2024**, *154*, 1169-1179, doi:10.1016/j.jaci.2024.06.022.
15. Yaakoubi, R.; Mekki, N.; Ben-Mustapha, I.; Ben-Khemis, L.; Bouaziz, A.; Ben Fraj, I.; Ammar, J.; Hamzaoui, A.; Turki, H.; Boussofara, L.; et al. Diagnostic challenge in a series of eleven patients with hyper IgE syndromes. *Frontiers in immunology* **2022**, *13*, 1057679, doi:10.3389/fimmu.2022.1057679.
16. Lachover-Roth, I.; Lagovsky, I.; Shtorch-Asor, A.; Confino-Cohen, R.; Reinstein, E.; Garty, B.Z. Hyper IgE Syndrome in an Isolated Population in Israel. *Frontiers in immunology* **2022**, *13*, 829239, doi:10.3389/fimmu.2022.829239.
17. Lan, J.; Zhang, Y.; Song, M.; Cai, S.; Luo, H.; OuYang, R.; Yang, P.; Shi, X.; Long, Y.; Chen, Y. Omalizumab for STAT3 Hyper-IgE Syndromes in Adulthood: A Case Report and Literature Review. *Front Med (Lausanne)* **2022**, *9*, 835257, doi:10.3389/fmed.2022.835257.
18. Ma, Y.; Sun, Q.; Zhang, K.; Bai, L.; Du, L. High level of IgE in acute low-tone sensorineural hearing loss: A predictor for recurrence and Meniere Disease transformation. *Am J Otolaryngol* **2021**, *42*, 102856, doi:10.1016/j.amjoto.2020.102856.
19. Awad, R.; Kakaje, A. Hyper IgE syndrome (Job syndrome) in Syria: a case report. *Oxf Med Case Reports* **2020**, *2020*, omaa106, doi:10.1093/omcr/omaa106.
20. Borst, J.; Ma, L. Oral ulcerations in a patient with autosomal dominant hyper-IgE syndrome (AD-HIES). *BMJ case reports* **2020**, *13*, doi:10.1136/bcr-2020-236705.

21. Singh, A.; Mandal, A.; Seth, R. Hyper IgE syndrome: often a missed diagnosis. *Int J Contemp Pediatr* **2016**, *3*, 674-677, doi:10.18203/2349-3291.ijcp20161064.
22. Yanagimachi, M.; Ohya, T.; Yokosuka, T.; Kajiwara, R.; Tanaka, F.; Goto, H.; Takashima, T.; Morio, T.; Yokota, S. The Potential and Limits of Hematopoietic Stem Cell Transplantation for the Treatment of Autosomal Dominant Hyper-IgE Syndrome. *J Clin Immunol* **2016**, *36*, 511-516, doi:10.1007/s10875-016-0278-1.
23. Patel, N.C.; Gallagher, J.L.; Torgerson, T.R.; Gilman, A.L. Successful haploidentical donor hematopoietic stem cell transplant and restoration of STAT3 function in an adolescent with autosomal dominant hyper-IgE syndrome. *J Clin Immunol* **2015**, *35*, 479-485, doi:10.1007/s10875-015-0167-z.
24. Purkait, R.; Kar, S.; Bhadra, R.; Sinhamahapatra, T. Acute disseminated encephalomyelitis: an uncommon presentation of hyper IgE syndrome. *Journal of the College of Physicians and Surgeons--Pakistan : JCPSP* **2014**, *24 Suppl 3*, S256-258.
25. Chandesris, M.O.; Melki, I.; Natividad, A.; Puel, A.; Fieschi, C.; Yun, L.; Thumerelle, C.; Oksenhendler, E.; Boutboul, D.; Thomas, C.; et al. Autosomal dominant STAT3 deficiency and hyper-IgE syndrome: molecular, cellular, and clinical features from a French national survey. *Medicine* **2012**, *91*, e1-e19, doi:10.1097/MD.0b013e31825f95b9.
26. Goussetis, E.; Peristeri, I.; Kitra, V.; Traeger-Synodinos, J.; Theodosaki, M.; Psarra, K.; Kanariou, M.; Tzortzatou-Stathopoulou, F.; Petrakou, E.; Fylaktou, I.; et al. Successful long-term immunologic reconstitution by allogeneic hematopoietic stem cell transplantation cures patients with autosomal dominant hyper-IgE syndrome. *J Allergy Clin Immunol* **2010**, *126*, 392-394, doi:10.1016/j.jaci.2010.05.005.
27. Joshi, A.Y.; Iyer, V.N.; Boyce, T.G.; Hagan, J.B.; Park, M.A.; Abraham, R.S. Elevated serum immunoglobulin E (IgE): when to suspect hyper-IgE syndrome-A 10-year pediatric tertiary care center experience. *Allergy Asthma Proc* **2009**, *30*, 23-27, doi:10.2500/aap.2009.30.3193.
28. Sarmento, K.M., Jr.; Tomita, S.; Caliman e Gurgel, J.D. Association between nasal polyposis, Dubowitz syndrome and hyper-IgE syndrome. *Int J Pediatr Otorhinolaryngol* **2008**, *72*, 711-714, doi:10.1016/j.ijporl.2008.01.018.
29. Freeman, A.F.; Kleiner, D.E.; Nadiminti, H.; Davis, J.; Quezado, M.; Anderson, V.; Puck, J.M.; Holland, S.M. Causes of death in hyper-IgE syndrome. *J Allergy Clin Immunol* **2007**, *119*, 1234-1240, doi:10.1016/j.jaci.2006.12.666.

30. Holland, S.M.; DeLeo, F.R.; Elloumi, H.Z.; Hsu, A.P.; Uzel, G.; Brodsky, N.; Freeman, A.F.; Demidowich, A.; Davis, J.; Turner, M.L.; et al. STAT3 mutations in the hyper-IgE syndrome. *N Engl J Med* **2007**, *357*, 1608-1619, doi:10.1056/NEJMoa073687.
31. DeWitt, C.A.; Bishop, A.B.; Buescher, L.S.; Stone, S.P. Hyperimmunoglobulin E syndrome: two cases and a review of the literature. *J Am Acad Dermatol* **2006**, *54*, 855-865, doi:10.1016/j.jaad.2005.10.022.
32. Moin, M.; Farhoudi, A.; Movahedi, M.; Rezaei, N.; Pourpak, Z.; Yeganeh, M.; Gharagozlou, M.; Mirsaeid Ghazi, B.; Arshi, S.; Mansouri, D.; et al. The clinical and laboratory survey of Iranian patients with hyper-IgE syndrome. *Scand J Infect Dis* **2006**, *38*, 898-903, doi:10.1080/00365540600740470.
33. Kimura, A.; Yoshino, H.; Yuasa, T. Chronic inflammatory demyelinating polyneuropathy in a patient with hyperIgEaemia. *J Neurol Sci* **2005**, *231*, 89-93, doi:10.1016/j.jns.2004.12.012.
34. Renner, E.D.; Puck, J.M.; Holland, S.M.; Schmitt, M.; Weiss, M.; Frosch, M.; Bergmann, M.; Davis, J.; Belohradsky, B.H.; Grimbacher, B. Autosomal recessive hyperimmunoglobulin E syndrome: a distinct disease entity. *The Journal of pediatrics* **2004**, *144*, 93-99, doi:10.1016/S0022-3476(03)00449-9.
35. Bilora, F.; Petrobelli, F.; Boccioletti, V.; Pomerri, F. Moderate-dose intravenous immunoglobulin treatment of Job's syndrome. Case report. *Minerva Med* **2000**, *91*, 113-116.
36. Grimbacher, B.; Schaffer, A.A.; Holland, S.M.; Davis, J.; Gallin, J.I.; Malech, H.L.; Atkinson, T.P.; Belohradsky, B.H.; Buckley, R.H.; Cossu, F.; et al. Genetic linkage of hyper-IgE syndrome to chromosome 4. *Am J Hum Genet* **1999**, *65*, 735-744, doi:10.1086/302547.
37. Fernandez, M.; Roman, J.; Latasa, M.; Oehling, A. Perforation of the nasal wall and hyper-IgE syndrome. *J Investig Allergol Clin Immunol* **1993**, *3*, 217-220.
38. AlYafie, R.; Velayutham, D.; van Panhuys, N.; Jithesh, P.V. The genetics of hyper IgE syndromes. *Frontiers in immunology* **2025**, *16*, 1516068, doi:10.3389/fimmu.2025.1516068.
39. Salehi, M.; Neshati, Z.; Ahanchian, H.; Tafrishi, R.; Pasdar, A.; Safi, M.; Karimiani, E.G. Hyper IgE Syndromes: Understanding, Management, and Future Perspectives: A Narrative Review. *Health Sci Rep* **2025**, *8*, e70497, doi:10.1002/hsr2.70497.
40. Sutanto, H.; Adytia, G.J.; Fetarayani, D. Hyper IgE Syndrome: Bridging the Gap Between Immunodeficiency, Atopy, and Allergic Diseases.

Curr Allergy Asthma Rep **2025**, *25*, 17, doi:10.1007/s11882-025-01196-8.

41. Gharehzadehshirazi, A.; Amini, A.; Rezaei, N. Hyper IgE syndromes: A clinical approach. *Clinical immunology (Orlando, Fla.)* **2022**, *237*, 108988, doi:10.1016/j.clim.2022.108988.
42. Minegishi, Y. Hyper-IgE syndrome, 2021 update. *Allergol Int* **2021**, *70*, 407-414, doi:10.1016/j.alit.2021.07.007.
43. Tsilifis, C.; Freeman, A.F.; Gennery, A.R. STAT3 Hyper-IgE Syndrome-an Update and Unanswered Questions. *J Clin Immunol* **2021**, *41*, 864-880, doi:10.1007/s10875-021-01051-1.
44. Tavakol, M.; Sharafian, S.; Salari, F.; Shokri, S. A Review on Hyper-IgE Syndromes, Clinical Manifestations, Diagnosis and Therapeutic Approaches. *Immunol Genet J* **2019**, *2*, 8-18, doi:10.22034/igj.2019.199870.1021.
45. Devilliers, H.; Turcu, A.; Vernier, N.; Muller, G.; Bielefeld, P.; Bonniaud, P.; Besancenot, J.F. [Hyper-IgE in internal medicine]. *Rev Med Interne* **2018**, *39*, 332-338, doi:10.1016/j.revmed.2017.12.009.
46. Ponsford, M.J.; Klocperk, A.; Pulvirenti, F.; Dalm, V.; Milota, T.; Cinetto, F.; Chovancova, Z.; Rial, M.J.; Sediva, A.; Litzman, J.; et al. Hyper-IgE in the allergy clinic--when is it primary immunodeficiency? *Allergy* **2018**, *73*, 2122-2136, doi:10.1111/all.13578.
47. Hashemi, H.; Mohebbi, M.; Mehravaran, S.; Mazloumi, M.; Jahanbani-Ardakani, H.; Abtahi, S.H. Hyperimmunoglobulin E syndrome: Genetics, immunopathogenesis, clinical findings, and treatment modalities. *J Res Med Sci* **2017**, *22*, 53, doi:10.4103/jrms.JRMS_1050_16.
48. Milner, J.D.; Sandler, N.G.; Douek, D.C. Th17 cells, Job's syndrome and HIV: opportunities for bacterial and fungal infections. *Curr Opin HIV AIDS* **2010**, *5*, 179-183, doi:10.1097/COH.0b013e328335ed3e.
49. Freeman, A.F.; Holland, S.M. Clinical manifestations, etiology, and pathogenesis of the hyper-IgE syndromes. *Pediatr Res* **2009**, *65*, 32R-37R, doi:10.1203/PDR.0b013e31819dc8c5.
50. Freeman, A.F.; Holland, S.M. The hyper-IgE syndromes. *Immunology and allergy clinics of North America* **2008**, *28*, 277-291, viii, doi:10.1016/j.iac.2008.01.005.
51. Hsu, A.P.; Davis, J.; Puck, J.M.; Holland, S.M.; Freeman, A.F. STAT3 Hyper IgE Syndrome. In *GeneReviews((R))*, Adam, M.P., Feldman, J., Mirzaa, G.M., Pagon, R.A., Wallace, S.E., Amemiya, A., Eds.; Seattle (WA), 1993.
